# Unilateral clubfoot with amniotic band in-utero

**DOI:** 10.11604/pamj.2021.39.169.30215

**Published:** 2021-07-06

**Authors:** Prerna Anup Patwa, Gaurav Vedprakash Mishra

**Affiliations:** 1 Department of Radiodiagnosis, Jawaharlal Nehru Medical College, Datta Meghe Institute of Medical Sciences, Sawangi (Meghe), Wardha, India

**Keywords:** Clubfoot, amniotic band, congenital talipes equinovarus

## Image in medicine

A 28-year-old primigravida with 22.3 weeks of gestation came for dedicated level II ultrasonography for anomaly scan to the antenatal clinic of AVBRH, Sawangi (India). The previous antenatal check-up was not done. The patient neither had a history of maternal systemic diseases like diabetes or hypertension nor history of any trauma or drug intake during pregnancy. There was no family history of congenital anomalies. On obstetric examination, the uterus was of about 20 weeks in size. On ultrasonography, single live intrauterine foetus with an average gestational age of 21 weeks 5 days corresponding to effective foetal weight of 396 grams showing right foot abnormality with persistent adduction and inward rotation suggestive of clubfoot with mild pylectasis of left kidney was noted. However, there was no obvious abnormality of left foot. There was amniotic band in right lower quadrant of gravid uterus.

**Figure 1:**
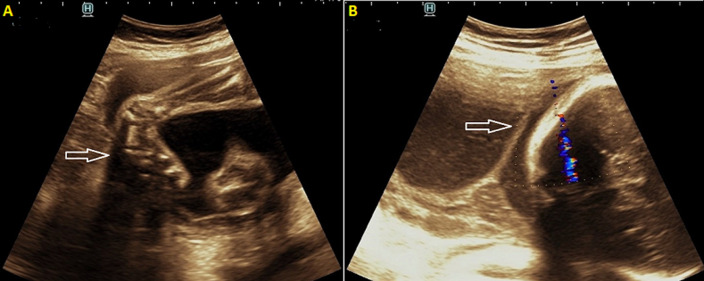
(A) ultrasound image showing medially rotated right foot suggesting club foot/CTEV; (B) ultrasound image showing a linear echogenic band at the lower end of uterus - amniotic band with an intact chorionic membrane

